# Repeat kidney biopsy in pediatric lupus nephritis: an analysis of associated clinical-pathological features and outcomes

**DOI:** 10.3389/fped.2026.1767283

**Published:** 2026-04-15

**Authors:** Wenyan Wang, Fei Zhao, Guixia Ding, Sanlong Zhao, Guoliang Ma, Xueqin Cheng

**Affiliations:** 1 Department of Nephrology, Children’s Hospital of Nanjing Medical University, Nanjing, Jiangsu, China; 2 Nanjing Municipal Center for Disease Control and Prevention, Nanjing, Jiangsu, China

**Keywords:** lupus nephritis (LN), pathology, pediatric, repeat kidney biopsy, treatment adjustment

## Abstract

**Background:**

The utility of repeat kidney biopsy in pediatric lupus nephritis (LN) remains debated. This study aims to evaluate pathological evolution, therapeutic implications, and prognostic impact of repeat biopsies in children with LN.

**Methods:**

We conducted a retrospective analysis of 19 pediatric LN patients (aged 5–18 years) who underwent ≥2 kidney biopsies at Nanjing Children's Hospital (2009–2022). Pathological classification (ISN/RPS criteria), activity/chronicity indices, and treatment responses were compared between biopsies. Statistical analyses were performed using SPSS 21.0, with *P* < 0.05 considered significant.

**Results:**

The primary indication for repeat biopsy was proteinuria recurrence (73.7%, 14/19). Pathological class transformation occurred in 47.4% (9/19) of cases (e.g., class III to IV), with a higher proportion of non-nephrotic type presentation in the transformation group (77.8% vs. 20.0%, *P* < 0.05). Treatment regimens were adjusted in 63.2% (12/19) of patients post-biopsy (e.g., cyclophosphamide to mycophenolate mofetil). Although chronicity scores increased significantly (e.g., glomerulosclerosis: 57.9% to 89.5%, *P* < 0.05), short-term kidney remission rates did not differ between groups with and without pathological transformation (77.8% vs. 80.0%, *P* > 0.05).

**Conclusion:**

Repeat kidney biopsy detects pathological progression (e.g., increased chronicity) and guides treatment modifications in over 60% of pediatric LN cases. However, pathological class change alone does not dictate short-term outcomes, highlighting initial treatment response as a stronger prognostic indicator. Individualized use of repeat biopsies is recommended for children with proteinuria relapse to optimize management while mitigating procedural invasiveness.

## 1 Introduction

Systemic lupus erythematosus (SLE) is a chronic autoimmune disorder characterized by multisystem involvement, and lupus nephritis (LN) represents one of its most severe complications, occurring in approximately 30%–60% of pediatric SLE patients (1–5). LN is a leading cause of morbidity and mortality in children, with studies indicating that kidney involvement develops earlier and progresses more rapidly in pediatric cohorts compared to adults. The pathogenesis of LN involves complex interactions between genetic, environmental, and immunological factors, leading to loss of self-tolerance, autoantibody production, immune complex deposition, and subsequent inflammation and damage to kidney tissues. Clinically, LN manifests with a spectrum of features, ranging from asymptomatic hematuria or proteinuria to nephrotic syndrome, acute kidney injury, and end-stage kidney disease (ESKD). Pediatric LN often presents with more aggressive disease activity, including higher rates of proliferative nephritis (International Society of Nephrology/Renal Pathology Society [ISN/RPS] Class III/IV), which is associated with poor outcomes if not promptly and adequately treated (6–9). Epidemiological data highlight that children with LN have a higher risk of progressing to ESRD, with 5%–20% developing kidney failure within 10 years of diagnosis. The management of pediatric LN requires early diagnosis and aggressive immunosuppressive therapy to induce and maintain remission, but long-term outcomes remain suboptimal due to disease flares, treatment-related toxicities, and cumulative organ damage (10–16).

Kidney biopsy is the gold standard for diagnosing and classifying LN, providing critical information on histopathological patterns that guide treatment decisions and prognosis assessment. According to the ISN/RPS classification system, LN is categorized into six classes (I-VI) based on light microscopy, immunofluorescence, and electron microscopy findings, with activity and chronicity indices quantifying inflammatory and fibrotic changes, respectively. The initial biopsy helps differentiate between proliferative (Class III/IV) and non-proliferative (Class V) forms, which require distinct therapeutic approaches; for instance, proliferative LN often necessitates aggressive immunosuppression with corticosteroids and cyclophosphamide or mycophenolate mofetil (17–19). However, LN is a dynamic disease, and pathological features can evolve over time due to treatment responses, flares, or chronic damage accumulation. Repeat kidney biopsy, performed for indications such as persistent proteinuria, unexplained kidney dysfunction, offers insights into these changes. Studies have shown that repeat biopsies reveal class switching in up to 50%–60% of cases, with proliferative LN potentially transitioning to mixed or membranous patterns, and vice versa. This histological evolution can significantly impact management, as it may necessitate therapy intensification or de-escalation. For example, an increase in chronicity index on repeat biopsy correlates with irreversible scarring and poorer long-term outcomes, guiding decisions on maintenance therapy. Meta-analyses and cohort studies underscore the utility of repeat biopsies in detecting subclinical activity, assessing treatment efficacy, and predicting kidney survival, particularly in pediatric populations where rapid progression is common. Despite its invasive nature, the procedural risks are generally low, and the histopathological data from repeat biopsies are beneficial for adjusting personalized treatment plans (2, 20, 21).

This study aims to investigate the clinical and pathological characteristics and outcomes associated with repeat kidney biopsies in pediatric patients with LN, addressing a gap in literature where most evidence derives from adult populations. Specifically, we seek to analyze changes in histopathological classes, activity and chronicity indices, and laboratory parameters between initial and subsequent biopsies, and correlate these with treatment responses and long-term kidney survival. By examining a cohort of children who underwent multiple biopsies, we will identify factors predictive of pathological transformation, such as shifts from proliferative to non-proliferative patterns or increases in chronic damage, and assess how these changes influence therapeutic strategies and remission rates. Additionally, we will evaluate the indications for repeat biopsy, including disease flare or resistance, and determine the proportion of cases where biopsy results led to modified immunosuppressive regimens. The prognosis will be gauged through endpoints like complete or partial kidney response, progression to chronic kidney disease stages, and need for kidney replacement therapy. Previous research, including meta-analyses and single-center reports, suggests that repeat biopsies can unveil discordances between clinical and histological remission, highlighting the importance of pathological monitoring in optimizing care. However, pediatric-specific data are limited, and this study will contribute insights into the natural history of LN in children, potentially informing guidelines on the timing and utility of repeat biopsies. Ultimately, our findings may help stratify patients based on biopsy-driven risk profiles, enabling tailored interventions to improve outcomes in this vulnerable population (12, 20, 21).

## 2 Methods and materials

### 2.1 Study population

A universal sampling method was used in this study.We retrospectively collected data from pediatric patients with lupus nephritis (LN) who were initially diagnosed in the Department of Nephrology at Nanjing Children's Hospital between December 2009 and December 2022,and who subsequently underwent two or more repeat kidney biopsies during follow-up treatment. Inclusion criteria were as follows: (1) patients were over 5 years old and had undergone at least two kidney biopsies; (2) all patients met the diagnostic criteria outlined in the “Evidence-Based Guidelines for the Diagnosis and Treatment of Lupus Nephritis (2016)” published by the Pediatric Branch of the Chinese Medical Association (11, 22). Exclusion criteria included: (1) patients lost to follow-up; (2) patients with incomplete clinical and pathological data.

### 2.2 Data collection

Clinical data were obtained through the electronic medical record system, covering the following aspects: basic information (e.g., gender, age at first onset), initial clinical symptoms, laboratory test results (including urinary protein, urinary red blood cells, 24-hour urinary protein quantification, complete blood count, blood urea nitrogen, creatinine, complement C3 and C4 levels), as well as pathological classification and treatment regimens. Follow-up was conducted via telephone and outpatient reviews, with the cutoff date set for June 2023.

### 2.3 Kidney pathological diagnosis and histological scoring

All kidney biopsy specimens were independently evaluated for pathological classification by two experienced pathologists at the associate professor level or above, who were blinded to the patients' clinical data. In cases of disagreement, a consensus was reached through discussion or by adjudication from a third, more senior pathologist. The assessment of lupus nephritis pathological classification (or specific lesions) by the two pathologists demonstrated good to excellent interobserver agreement (Kappa = 0.8; 95% CI 0.7–0.9). The classification of kidney pathology followed the 2003 International Society of Nephrology/Renal Pathology Society (ISN/RPS) revised classification criteria [3]. All kidney tissue specimens underwent routine procedures including direct immunofluorescence, light microscopy, and electron microscopy. Scoring was based on the lupus nephritis activity index and chronicity index criteria established by the National Institutes of Health (23).

### 2.4 Lupus nephritis clinical classification

In this study, patients were clinically classified into two types based on the level of proteinuria. Those presenting with massive proteinuria, defined as 24 h urine protein quantification ≥50 mg/kg, were classified as the nephrotic type, while those without massive proteinuria (24 h urine protein quantification <50 mg/kg) were defined as the nephritic type.

### 2.5 Treatment protocol

Treatment Protocol All patients were administered glucocorticoid therapy, specifically prednisone acetate at a dose of 2 mg/(kg·d), with subsequent tapering based on clinical response. The selection of immunosuppressive agents during the induction phase was guided by the following protocol: ① Based on kidndy biopsy histopathology, clinical presentation, and the Systemic Lupus Erythematosus Disease Activity Index (SLEDAI) score, patients with class I or II lupus nephritis (LN) and without severe manifestations (e.g., central nervous system involvement) received glucocorticoids in combination with hydroxychloroquine sulfate. Patients with class III, IV, or V LN were treated with either intravenous cyclophosphamide (CTX) pulse therapy (10–20 mg/kg per dose) every two weeks to one month, or oral mycophenolate mofetil (MMF) at a dose of 1,200 mg/m² per day, divided into two administrations. Following the achievement of remission in the induction phase, maintenance therapy was continued with either CTX pulses, MMF, or azathioprine. In cases of disease relapse during maintenance treatment, the therapeutic agent was selected according to findings from a repeat kidney biopsy or by re-instituting the induction regimen that had proven effective initially. If remission was not attained after 6 months of induction therapy, or if complete remission was not achieved within approximately one year, the regimen was switched from CTX or MMF to either cyclosporine (3–6 mg/(kg·d)), tacrolimus (0.10–0.15 mg/(kg·d)), or a multi-target combination therapy.

### 2.6 Concomitant medications

Concomitant medications were administered in a standardized manner to all patients in addition to the core immunosuppressive regimen, in strict accordance with relevant clinical practice guidelines and the institutional treatment protocol, for the management of pre-existing comorbidities and the mitigation of treatment-related adverse reactions. Hydroxychloroquine was prescribed to all patients in the absence of contraindications, at a target dose of 5 mg/(kg·d) with a maximum daily dose capped at 400 mg. For all pediatric patients, routine supplementation with vitamin D and calcium carbonate was implemented to prevent and manage glucocorticoid-induced osteoporosis and support normal growth and development, with individualized dose adjustments based on age, body weight, and serum 25-hydroxyvitamin D levels. For kidney protection, all patients with documented proteinuria received either an angiotensin-converting enzyme inhibitor (ACEI) or an angiotensin II receptor antagonist (ARB): benazepril hydrochloride was initiated at 0.1–0.2 mg/(kg·d) (maximum daily dose 15 mg), and valsartan was initiated at 1–2 mg/(kg·d) (maximum daily dose 160 mg), with subsequent dose titration based on serial blood pressure monitoring and treatment tolerability. Additionally, dipyridamole was added to the therapeutic regimen for patients with persistent proteinuria, hypercoagulable state, or kidney biopsy findings indicative of thrombotic microangiopathy or vascular lesions, at a dose of 3–5 mg/(kg·d) and a maximum daily dose of 150 mg.

### 2.7 Efficacy evaluation

Treatment response was evaluated at 6 months after the initiation of therapy. Treatment efficacy was assessed according to the 2012 Kidney Disease: Improving Global Outcomes (KDIGO) guidelines (24), covering three aspects: complete remission, partial remission, and no response to treatment. Complete remission was defined as urinary protein levels below 0.5 g/24 h, with estimated glomerular filtration rate (eGFR) maintained at normal or near-normal levels; partial remission referred to a reduction in urinary protein by more than 50% from baseline but not exceeding 3.5 g/24 h, with eGFR maintained normal or near-normal; no response indicated failure to meet the criteria for complete or partial remission. End-stage kidney disease (ESKD) is defined as a sustained estimated glomerular filtration rate below a specified threshold (thereby excluding cases of acute kidney injury). Non-adherence to treatment was defined as follows: ① during cyclophosphamide (CTX) treatment, if the interval between pulse therapies exceeded 45 days or the course was less than 6 sessions, it was considered non-adherence; ② during mycophenolate mofetil (MMF) treatment, if the child self-discontinued the drug for more than one week based on parental report, it was deemed medication non-adherence; ③ the definition of non-adherence for other immunosuppressants (including hormones) was the same as for MMF.

### 2.8 Systemic lupus erythematosus disease activity index scoring

The Systemic Lupus Erythematosus Disease Activity Index (SLEDAI) was scored according to the 2000 scoring criteria (25), specifically categorized as: 0–4 points indicating minimal activity; 5–9 points indicating mild activity; 10–14 points indicating moderate activity; and ≥15 points indicating severe activity.

### 2.9 Statistical analysis

All data were statistically analyzed using SPSS software version 21.0. For continuous data, the Shapiro–Wilk test was used to assess normal distribution; if *P* > 0.05, the data were considered to follow a normal distribution. Normally distributed continuous data are presented as mean ± standard deviation (x ± s). Comparisons between two groups were analyzed using Student's *t*-test. Qualitative data are expressed as frequencies and percentages (%), and Fisher's exact test or chi-square test was used for comparisons between two groups. A *P*-value < 0.05 was considered statistically significant.

## 3 Results

### 3.1 Analysis of indications for repeat kidney biopsy

A total of 21 pediatric patients underwent two or more kidney biopsies. After excluding one patient lost to follow-up and another with incomplete clinical data, 19 patients were included in the final analysis. For each included patient, data from the first two biopsies were selected for evaluation. Among the 19 pediatric patients who underwent repeat kidney biopsies, 14 cases (73.7%) underwent repeat biopsies due to disease relapse with recurrent proteinuria during maintenance therapy. The detailed clinical manifestations of these 14 relapsed patients were further characterized as follows: 8 cases presented with hematological system involvement marked by anemia, thrombocytopenia and hypocomplementemia, concurrent with recurrent proteinuria; 3 cases manifested as fever combined with hypocomplementemia and recurrent proteinuria; 2 cases developed recurrent proteinuria during the tapering of maintenance immunosuppressive therapy; and 1 case presented with recurrent facial rash accompanied by recurrent proteinuria. Another 4 cases (21.1%) underwent repeat biopsies for persistent proteinuria unresponsive to initial treatment, with the aim of adjusting subsequent therapeutic strategies. One patient (5.3%) required a repeat biopsy due to mild proteinuria accompanied by kidney tubular acidosis and persistent hypokalemia during ongoing treatment (Table 1).Procedure-related complications were minimal, with no bleeding events detected after the first kidney biopsy. Following repeat kidney biopsy, one patient developed mild bleeding, which was successfully managed with conservative treatment involving intravenous carbazochrome sodium sulfonate, and the patient achieved rapid clinical recovery thereafter.

**Table 1:** Clinical characteristics and treatment regimens of 19 pediatric lupus nephritis patients undergoing repeat kidney biopsies.

ID	Age, years	Interval, months	Gender	Clinical type	SLDAI score	Pathology, first biopsy	Initial treatment regimen	Outcome	Pathology, second biopsy	Reason for repeat kidney biopsy	Post-biopsy outcome	Kidney function	Change in Treatment Plan, Yes/No
1	14.1	13	Female	Non-nephrotic	14	Class Ⅱ	Pred	CR	Class Ⅱ	Relapse	CR	CKD1	Yes
2	11.6	25	Female	Non-nephrotic	16	Class Ⅳ	Pred + CTX	CR	Class Ⅲ + Ⅴ	Relapse	CR	CKD1	Yes
3	5.5	85	Female	Nephrotic	8	Class Ⅳ	Pred + CTX	CR	Class Ⅱ	Relapse	CR	CKD1	No
4	8.9	14	Male	Non-nephrotic	21	Class Ⅲ + Ⅴ	Pred + CTX	NR	Class Ⅲ + Ⅴ	NR	NR	CKD1	Yes
5	9.6	16	Female	Non-nephrotic	17	Class Ⅳ	Pred + CTX	CR	Class Ⅳ + Ⅴ	Relapse	NR	CKD5	Yes
6	5.8	26	Female	Nephrotic	19	Class Ⅲ	Pred + CTX	CR	Class Ⅳ + Ⅴ	Relapse	CR	CKD1	Yes
7	10.9	25	Male	Non-nephrotic	18	Class Ⅴ	Pred + CsA	CR	Class Ⅴ	Relapse	CR	CKD1	Yes
8	9.6	17	Female	Nephrotic	23	Class Ⅳ + TMA	Pred + CTX	NR	Class Ⅳ + TMA	NR	NR	CKD5	Yes
9	9.8	70	Male	Nephrotic	15	Class Ⅳ	Pred + CTX	CR	Class Ⅳ + Ⅴ + TMA	Relapse	CR	CKD5	Yes
10	10.6	63	Male	Non-nephrotic	13	Class Ⅳ	Pred + CTX	CR	Class Ⅳ + Ⅴ	Relapse	CR	CKD1	Yes
11	9.7	87	Female	Nephrotic	17	Class Ⅳ	Pred + MMF	CR	Class Ⅳ	Relapse	CR	CKD1	No
12	8.6	37	Female	Nephrotic	19	Class Ⅳ	Pred + CTX	CR	Class Ⅳ + TMA	Relapse	CR	CKD1	Yes
13	13.5	15	Male	Nephrotic	20	Class Ⅳ	Pred + CTX	NR	Class Ⅳ	NR	NR	CKD2	No
14	11.7	38	Male	Nephrotic	24	Class Ⅳ + Ⅴ	Pred + MMF	NR	Class Ⅳ + Ⅴ + TMA	NR	NR	CKD5	No
15	9.8	68	Male	Nephrotic	22	ClassⅣ	Pred + CTX	CR	ClassⅣ	Relapse	CR	CKD1	Yes
16	10.7	46	Female	Nephrotic	19	ClassⅣ	Pred + CTX	CR	ClassⅣ	Relapse	CR	CKD1	No
17	7.6	61	Female	Non-nephrotic	17	ClassⅢ	Pred + MMF	CR	ClassⅣ	Relapse	CR	CKD1	Yes
18	11.6	37	Female	Non-nephrotic	21	ClassⅤ	Pred + CTX	CR	ClassⅢ + Ⅴ	RTA	CR	CKD1	No
19	10.4	43	Female	Non-nephrotic	17	ClassⅣ	Pred + CTX	CR	ClassⅣ	Relapse	CR	CKD1	No

Pred, prednisone; CTX, cyclophosphamide; MMF, mycophenolate mofetil; CsA, cyclosporine; CR, complete remission; NR, no remission); RTA, renal tubular acidosis; CKD, chronic kidney disease.

Explanation of Table 1: This table summarizes the demographic, clinical, and pathological details of 19 pediatric LN patients who underwent repeat kidney biopsies. It highlights key aspects such as age, biopsy intervals, clinical types, SLEDAI scores, pathological classifications at both biopsies, reasons for repeat procedures, treatment adjustments, and outcomes. The data reveal that disease relapse and persistent proteinuria were the primary indications for repeat biopsies, leading to therapy modifications in most cases. The table underscores the dynamic nature of LN and the role of histopathological monitoring in guiding personalized treatment.

### 3.2 Clinical characteristics and outcomes

Among the 19 children with lupus nephritis, 6 were male and 13 were female. The age at disease onset ranged from 5 to 18 years, with a mean age at onset of 9.4 ± 3.1 years. Ten patients (52.6%) presented with nephrotic syndrome. Nine patients (47.4%) exhibited chronic nephritic syndrome, among them three presented with isolated proteinuria and six presented with hematuria accompanied by non-nephrotic range proteinuria. And we defined children presenting without nephrotic syndrome as the non-nephrotic type. Notably, one patient also had renal tubular acidosis. The disease duration ranged from 1 to 14 years at the end of follow-up. Based on the initial SLEDAI scores, 1 patient (5.3%) had mild activity, 2 (10.5%) had moderate activity, and 16 (84.2%) had severe activity. At the first biopsy, pathological findings included 12 cases of Class IV, 2 of Class III, 2 of Class V, and 1 each of Class III + V, IV + V, and II. After repeat biopsy, 9 patients (47.4%) showed changes in pathological class. Treatment regimens were adjusted in 12 patients (63.2%) following repeat biopsy. Following treatment after the initial kidney biopsy, complete remission was achieved in 15 patients (78.9%), while 4 patients (21.1%) showed no response. During follow-up, 4 patients progressed to CKD stage 5, and 1 was in CKD stage 2 (Table 1).

### 3.3 Comparison between pathological class change and non-change groups at second biopsy

Comparative analysis between the two groups showed a significantly higher proportion of patients with non-nephrotic type presentation in the pathological class change group (*P* < 0.05). However, no statistically significant differences were observed in gender, age, treatment adherence, initial induction therapy, hypertension incidence, or initial SLEDAI scores (*P* > 0.05). Increases in chronicity and activity scores were noted in both groups but without statistical significance (*P* > 0.05). Tubulointerstitial damage was more frequent in the change group, though the difference was not significant (*P* > 0.05). Kidney remission rates did not differ significantly between groups (*P* > 0.05) (Table 2).

**Table 2:** Comparison between patients with and without pathological class change at second kidney biopsy.

Variables	Pathological type change group (*n* = 9)	Pathological Type non-change group (*n* = 10)	Statistic	*P*-value
Demographics				
Age (mean ± SD), years	9.2 ± 2.4	11.12 ± 1.9	*t* = 0.6	0.4
Gender, male/female	2/7	4/6	*χ^2^* = 0.7	0.4
Clinical Features, *n* (%)				
Non-nephrotic type	7 (77.8)*	2 (20.0)	*χ^2^* = 6.7	0.0
Nephrotic type	2 (22.2)	8 (80.0)		
Hypertension, *n* (%)	4 (44.4)	3 (30.0)	*χ^2^* = 0.4	0.5
Compliance, *n* (%)				
Good compliance	6 (66.7)	8 (80.0)	*χ^2^* = 0.4	0.5
Poor compliance	3 (33.3)	2 (20.0)		
Initial SLEDAI score, *n* (%)				
Mild to moderate	2 (22.2)	3 (30.0)	*χ^2^* = 0.1	0.7
Severe	7 (77.8)	7 (70.0)		
Initial treatment regimen, *n* (%)				
Pred + CTX	6 (66.7)	4 (40.0)	*χ^2^* = 0.9	0.3
Pred + MMF	3 (33.3)	5 (50.0)		
Mean interval between repeat kidndy biopsies (mean ± SD), months	49.4 ± 26.9	31.0 ± 19.8	*t* = 1.7	0.1
Repeat Biopsy and Pathological Progression, *n* (%)				
Cases with increased chronicity score on second biopsy, *n* (%)	4 (44.4)	4 (40.0)	*χ^2^* = 0.0	0.8
Cases with increased activity score on second biopsy, *n* (%)	2 (22.2)	3 (30.0)	*χ^2^* = 0.1	0.7
Cases with worsened tubulointerstitial damage, *n* (%)	5 (55.6)	2 (20.0)	*χ^2^* = 2.6	0.1
Treatment Outcomes, *n* (%)				
Complete remission	7 (77.8)	8 (80.0)	*χ^2^* = 0.0	0.9
No remission	2 (22.2)	2 (20.0)		

Explanation of Table 2: This table compares demographic, clinical, and pathological parameters between patients with and without pathological class changes on repeat biopsy. It demonstrates that while non-nephrotic type presentation was more common in the change group, other baseline characteristics were similar. Compared with Non-Change Groups.

* *P* < 0.05.

### 3.4 Comparative analysis of clinical and pathological features before and after two biopsies in 19 LN patients

No significant differences were found in 24 h urine protein, complement C3 levels, hypertension, or hematuria between the two biopsies (*P* > 0.05). However, the incidence of cellular/fibrocellular crescents increased significantly (*P* < 0.05). Neutrophil infiltration/karyorrhexis and interstitial inflammation showed no significant changes (*P* > 0.05). For chronicity indices, glomerulosclerosis and tubular atrophy rates differed significantly (*P* < 0.05), but fibrous crescents and interstitial fibrosis did not (*P* > 0.05). The mean chronicity score increased significantly (*P* < 0.05), while the mean activity score remained unchanged (*P* > 0.05) (Table 3).

**Table 3:** Comparison of clinical and pathological features before and after Two kidney biopsies in 19 LN patients.

Variables	First kidney biopsy (*n* = 19)	Second kidney biopsy (*n* = 19)	Statistical value	*P*-value
Laboratory and Clinical Parameters				
24 h urinary protein (mean ± SD), g/d	1.2 ± 0.5	1.1 ± 0.5	*t* = −0.8	0.4
Complement C3 (mean ± SD), g/L	0.4 ± 0.2	0.4 ± 0.1	*t* = −0.4	0.7
Hematuria, *n* (%)	14 (73.7)	16 (84.2)	*F* = 0.6	0.7
Hypertension, *n* (%)	7 (36.8)	11 (57.9)	*F* = 1.7	0.2
SLEDAI score				
Mild to moderate, *n* (%)	3 (15.8)	5 (26.3)	*F* = 0.6	0.4
Severe, *n* (%)	16 (84.2)	14 (73.7)		
Activity index, *n* (%)				
Neutrophil infiltration and/or karyorrhexis	6 (31.6)	7 (36.8)	*F* = 0.1	0.7
Cellular and/or fibrocellular crescent formation	13 (68.4)	18 (94.7)*	*F* = 4.8	0.0
Interstitial inflammatory cell infiltration	15 (78.9)	16 (84.2)	*F* = 0.2	0.7
Chronicity index, *n* (%)				
Glomerulosclerosis	11 (57.9)	17 (89.5)*	*F* = 7.5	0.0
Fibrous crescent formation	13 (68.4)	16 (84.2)	*F* = 1.3	0.3
Interstitial fibrosis	5 (26.3)	10 (52.6)	*F* = 2.7	0.1
Tubular atrophy	3 (15.8)	10 (52.6)*	*F* = 5.9	0.0
Composite Scores				
Median activity score [M(P25–P75)], points	7 (6,9)	7 (6,9)	*Z* = 0.6	0.6
Median chronicity score [M(P25–P75)], points	1 (0,2)	3 (1,6)*	*Z* = −2.5	0.0
Outcome, *n* (%)				
Complete remission	15 (78.9)	14 (73.7)	*F* = 0.2	0.7
No remission	4 (21.1)	5 (26.3)		

Explanation of Table 3: This table provides a longitudinal comparison of clinical and pathological features between the first and repeat biopsies. It highlights a significant progression in chronic damage (e.g., glomerulosclerosis and tubular atrophy) despite stable clinical parameters, underscoring the value of repeat biopsies in detecting subclinical disease progression. The increase in cellular crescents suggests ongoing activity, which may inform treatment intensification.

*Compared with first kidney biopsy, *P* < 0.05.

### 3.5 Comparison of clinical outcomes between groups with and without increased chronicity scores

In this study, patients were stratified into a high-chronicity group and a low-chronicity group based on changes in the chronicity index following repeat kidney biopsy, and their clinical characteristics and outcomes were compared. No significant difference was observed in baseline serum creatinine between the two groups (*P* > 0.05). Similarly, the clinical remission rates after the initial kidney biopsy did not differ significantly (*P* > 0.05). However, by the end of follow-up, serum creatinine was significantly higher in the high-chronicity group compared to the low-chronicity group (*P* < 0.05). Although the clinical remission rate after repeat biopsy was lower in the high-chronicity group than in the low-chronicity group, this difference was not statistically significant (*P* > 0.05) (Table 4).

**Table 4:** Comparison of clinical outcomes between groups with and without increased chronicity scores.

Clinical outcomes	High-chronicity group (*n* = 12)	Low-chronicity group (*n* = 7)	Statistical value	*P*-value
Baseline Scr [M(P25–P75)]	52.0 (48.0–56.5)	49.0 (45.0–54.0)	U = 34.0	0.5
Remission rate after initial biopsy [*n*(%)]	9 (75)	7 (100)	F = 3.1	0.3
Non-remission rate after initial biopsy [*n*(%)]	3 (25)	0 (0)		
Remission rate after repeat biopsy [*n*(%)]	8 (66.7)	7 (100)	F = 4.3	0.2
Non-remission rate after repeat biopsy [*n*(%)]	4 (33.3)	0 (0)		
Follow-up Scr[M(P25–P75)]	70.5 (60.5–114.5)*	56.0 (52.0–63.0)	U = 13.0	0.0

Explanation of Table 4: The table compares clinical remission after initial and repeat biopsies between groups with and without increased chronicity scores. No significant difference in remission rates was observed after either biopsy. However, at follow-up end, serum creatinine was significantly higher in the high-chronicity group, suggesting that increased chronicity may relate to long-term changes in glomerular filtration rate.

* Compared with low-chronicity group, *P* < 0.05.

## 4 Discussion

The present study provides a comprehensive analysis of the role of repeat kidney biopsy in pediatric lupus nephritis (LN), demonstrating that procedural indications are primarily driven by persistent or relapsing proteinuria, with nearly half of patients exhibiting pathological class transformation upon re-evaluation. Our findings align with existing literature underscoring the dynamic nature of LN histology, where shifts in ISN/RPS classification—particularly from proliferative to mixed or membranous patterns—may reflect evolving disease activity or treatment response (21, 26). Notably, the increase in chronicity scores (e.g., glomerulosclerosis and tubular atrophy) on repeat biopsies highlights progressive kidney damage despite clinical remission, echoing observations by Morales et al. that chronic pathological changes often advance subclinically in pediatric LN (27). However, the lack of a significant difference in kidney outcomes between groups with and without pathological transformation suggests that class switching alone may not dictate prognosis, emphasizing the need for integrative assessment of both activity and chronicity indices. This resonates with guidelines recommending repeat biopsies for unresolved clinical questions, as histological data can uncover discordances between clinical and pathological remission (11, 24). As observed in our study, there was no significant difference in the SLEDAI scores between the initial and repeat kidney biopsies. However, the chronicity index of the kidney pathology was significantly higher in the repeat biopsy group. This suggests that although systemic disease activity did not markedly increase, the progression of kidney chronic fibrosis did not halt.

Proteinuria recurrence emerged as the predominant indication for repeat biopsy in our cohort (73.7%), consistent with prior studies identifying it as a sentinel marker of disease flare or resistance (28, 29). This aligns with recommendations by Nachman et al., who advocate for repeat biopsies in LN patients with persistent proteinuria to guide immunosuppression adjustments (28). Kajawo et al. similarly reported that over 85% of patients had therapy changes after repeat biopsy, highlighting its role in optimizing control of active disease (29).The study by Narváez J et al. also indicated that over half of the patients had their immunosuppressive treatment regimens adjusted after a repeat kidney biopsy, usually involving the addition of an immunosuppressant to the existing therapy (29). Our data further revealed that 63.2% of patients underwent treatment modifications following repeat biopsy. These adjustments included the addition of the immunosuppressant mycophenolate mofetil to corticosteroid-only regimens, the substitution of cyclophosphamide with tacrolimus or the initiation of multi-target therapy (tacrolimus plus mycophenolate mofetil) in non-responding patients, and the adjunctive use of therapeutic plasma exchange in three patients who developed thrombotic microangiopathy during mycophenolate mofetil maintenance therapy. Additionally, some patients discontinued mycophenolate mofetil and resumed cyclophosphamide, switched to tacrolimus, or received adjunctive CD20-targeted therapy. For children presenting with recurrent proteinuria, timely repeat kidney biopsy facilitates the assessment of changes in kidney activity and chronicity scores, thereby enabling precise adjustment of treatment strategies.

Pathological class changes occurred in 47.4% of patients, frequently involving transitions to mixed or proliferative patterns, which corroborates studies by Tannor et al. indicating that histological evolution is common in LN flares (30). It is noteworthy that, in the present study, a considerable proportion of newly emerged histopathological transformations involved class V lesions. However, regardless of whether pathological transformation occurred, there was no significant difference in kidney remission between the two groups of pediatric patients. Furthermore, a comparison of clinical characteristics between the histopathological transformation and non-transformation groups revealed that patients presenting with non-nephrotic type manifestations were more susceptible to pathological class change, with the majority developing newly emerging class V lesions. This suggests that although the clinical presentation of non-nephrotic type patients may be less severe than that of nephrotic type patients, cautious adjustment of the treatment regimen during the maintenance phase remains essential for this group to prevent potential worsening of pathological damage.

The significant rise in chronicity scores—particularly glomerulosclerosis and tubular atrophy—on repeat biopsies underscores the propensity for fibrotic damage accumulation over time, even amid clinical stability. This phenomenon aligns with work by Pagni et al., who identified tubulointerstitial fibrosis as an independent predictor of long-term kidney decline (31). In our cohort, patients were stratified into a high-chronicity group and a low-chronicity group based on changes in the chronicity index following repeat kidney biopsy. No significant difference was observed in baseline creatinine clearance or in clinical remission rates after the initial kidney biopsy between the two groups. However, by the end of follow-up, creatinine clearance was significantly higher in the high-chronicity group compared to the low-chronicity group. Although the clinical remission rate after repeat biopsy was lower in the high-chronicity group, this difference was not statistically significant. In our cohort, the increased incidence of cellular/fibrocellular crescents further suggests ongoing immune activity, potentially driven by unresolved immune complex deposition. Importantly, the dissociation between stable activity scores and worsening chronicity highlights the limitations of relying solely on clinical parameters; as Bajema et al. emphasize, chronicity indices provide prognostic insights beyond activity measures (23). For example, patients with elevated chronicity may benefit from prolonged maintenance therapy, even if clinically quiescent. These observations advocate for incorporating chronicity assessment into standard monitoring protocols, as timely identification of fibrosis may inform strategies to preserve kidney reserve.

Treatment modifications following repeat biopsy were implemented in most patients, yet the comparable remission rates across groups suggest that histological data alone may not suffice to alter outcomes without addressing underlying disease drivers. Our data show that initial treatment non-responders tended to remain resistant despite therapy adjustments, reinforcing the importance of achieving early remission as a prognostic marker, consistent with findings by Narvaez et al. (20). The lack of correlation between pathological transformation and ultimate kidney response implies that factors such as genetic predisposition, environmental triggers, and immunoregulatory mechanisms may outweigh biopsy findings in determining outcomes. Gupta et al. similarly noted that while rebiopsy guides therapy, long-term survival depends on multifaceted management, including comorbidity control and drug adherence (32). Looking ahead, future studies should explore non-invasive biomarkers (e.g., urinary chemokines or serum autoantibody profiles) to reduce biopsy reliance, particularly in children (33). Ultimately, our results underscore that repeat biopsies are a valuable adjunct in pediatric LN, but their utility is maximized when integrated with clinical vigilance and shared decision-making, aiming to balance risk mitigation with personalized care.

## 5 Conclusion

This study explored the role of repeat kidney biopsy in the management of pediatric lupus nephritis, especially for patients with recurrent proteinuria. Repeat kidney biopsy can effectively detect the progression of kidney chronic damage, and histological evidence from such biopsies has informed treatment modifications in clinical practice, even without a standardized adjustment protocol in the present study. Pathological class transformation was observed in pediatric cases, and elevated chronicity scores on repeat biopsy were predictive of short-term kidney functional outcomes, whereas pathological class transformation alone did not influence short-term kidney remission, highlighting initial treatment response as a key short-term prognostic factor. As a single-center retrospective study, this research requires validation by multicenter prospective trials. Nevertheless, the findings support the use of individualized repeat kidney biopsy for high-risk pediatric lupus nephritis patients with recurrent proteinuria, which clarifies the progression of chronic kidney damage to guide long-term kidney protection strategies, while balancing the risks of invasive procedures with their clinical benefits.

## Data Availability

The original contributions presented in the study are included in the article/Supplementary Material, further inquiries can be directed to the corresponding author/s.
